# Association between cannabis use with urological cancers: A population‐based cohort study and a mendelian randomization study in the UK biobank

**DOI:** 10.1002/cam4.5132

**Published:** 2022-08-17

**Authors:** Jingyi Huang, Da Huang, Xiaohao Ruan, Jinlun Huang, Danfeng Xu, Susan Heavey, Jonathan Olivier, Rong Na

**Affiliations:** ^1^ Department of Urology Ruijin Hospital, Shanghai Jiao Tong University School of Medicine Shanghai China; ^2^ Division of Surgery and Interventional Sciences University College London London UK; ^3^ Department of Urology, CHU Lille Claude Huriez Hospital, University of Lille Lille France; ^4^ Division of Urology, Department of Surgery Queen Mary Hospital, The University of Hong Kong Hong Kong

**Keywords:** bladder cancer, cannabis, incidence, prostate cancer, renal cell carcinoma, testicular cancer

## Abstract

**Background:**

Legislation of cannabis use has been approved in many European and North American countries. Its impact on urological cancers is unclear. This study was conducted to explore the association between cannabis use and the risk of urological cancers.

**Methods:**

We identified 151,945 individuals with information on cannabis use in the UK Biobank from 2006 to 2010. Crude and age‐standardized incidence ratios of different urological cancers were evaluated in the entire cohort and subgroups. Cox regression was performed for survival analysis.

**Results:**

Previous use of cannabis was a significant protective factor for renal cell carcinoma (HR = 0.61, 95%CI:0.40–0.93, *p* = 0.021) and prostate cancer (HR = 0.82, 95%CI:0.73–0.93, *p* = 0.002) in multivariable analysis. The association between previous cannabis use and both renal cell carcinoma and bladder cancer was only observed in females (HR_RCC_ = 0.42, 95%CI:0.19–0.94, *p* = 0.034; HR_BCa_ = 0.43, 95%CI:0.21–0.86, *p* = 0.018) but not in men. There was no significant association between cannabis use and testicular cancer incidence. Mendelian randomization demonstrated a potential causal effect of cannabis use on a lower incidence of renal cell carcinoma.

**Conclusions:**

Previous use of cannabis was associated with a lower risk of bladder cancer, renal cell carcinoma, and prostate cancer. The inverse association between cannabis and both renal cell carcinoma and bladder cancer was only found in females but not in males.

## Graphical abstract

Previous cannabis use had a inverse association with BCa, RCC and PCa. For BCa and RCC, the effect of cannabis use was significant in females but not in males.

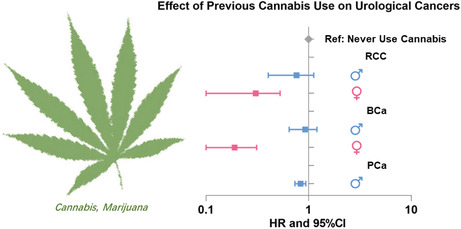

## 1 INTRODUCTION

Cannabis, also known as marijuana, is the most used substance derived from *Cannabis Sativa* which can be used for recreational or medical purposes.^1^ According to the World Drug Report 2021 published by the United Nations Office on Drugs and Crime, Canada, Uruguay and 20 jurisdictions in the United States have legalized the recreational use of cannabis since 2013.^1^ In 2019, roughly 200 million people were estimated consuming cannabis worldwide. C*annabis sativa* contains more than 500 components, including over 150 different kinds of cannabinoids,^2^ among which tetrahydrocannabinol (THC) is the major component with a psychoactive effect.^3^, ^4^

The use of cannabis is still controversial due to the lack of reliable evidence about its harm. It has a significant impact on the central nervous system. Psychotic disorders and cognitive defects are the main adverse effects of overdoses. Long‐term use of cannabis could be associated with respiratory and cardiovascular toxicity.^4^ On the contrary, medical cannabis is applied to deal with nausea, vomiting, anorexia, and chronic pain, especially for patients undergoing chemotherapy or palliative treatment for cancers.^5^ Some evidence also suggested that cannabinoids might induce apoptosis of cancer cells and inhibit oncogenesis, indicating a potential treatment effect, though this effect has only been observed in vitro.^2^, ^6^

To date, the association between cannabis and urological cancers remained unclear. Some studies suggested that current cannabis use might increase the risk of testicular cancer (TCa).^7^, ^8^ Unlike tobacco smoking which is widely acknowledged as an important risk factor for bladder cancer (BCa), the studies investigating the relationship between cannabis and BCa indicated contradictory results. For example, a case–control study conducted by Chacko et al. demonstrated a promoting effect of cannabis on transitional cell carcinoma of the bladder.^9^ While Thomas et al. concluded that cannabis use was associated with a lower incidence of BCa based on the California Men's Health Study cohort.^10^ Limited evidence was reported regarding the relationship between cannabis smoking and prostate cancer (PCa) or renal cell carcinoma (RCC). Several functional studies elaborated that cannabinoids appeared to inhibit cell proliferation, migration, and angiogenesis in urological cancer cells.^11^, ^12^, ^13^

Therefore, the present study aimed to evaluate the association between cannabis smoking and the incidence of urological cancers (BCa, RCC, PCa, and TCa) based on a prospective, population‐based cohort of the UK Biobank (UKB). The Mendelian Randomization (MR) approach was also applied to investigate the causal relationship between cannabis lifetime use and cancer risks.

## 2 PATIENTS AND METHODS

### 2.1 Study population

The UKB is a large‐scale biomedical database containing a prospective cohort with genetic and phenotype information in which approximately half a million UK participants aged between 40 and 69 years were recruited from 2006 to 2010.^14^ The present study is based on the latest follow‐up updated in May 2020. Cannabis use (ever, never, etc.) and tobacco smoking were defined based on the standard questionnaire of the study cohort. Tobacco smoking was considered one of the most important confounding factors when determining the effect of cannabis use in medical studies.^10^ Exclusion criteria include: (1) cases with the unknown diagnostic status of BCa, RCC, PCa, and TCa; (2) cases with uncertain cannabis or tobacco use history. Only Caucasian participants were included in the present study due to the limited number of other ethnicities.

### 2.2 Demographic and clinical information

Demographic characteristics including race/origin, gender, body mass index (BMI), and recruitment age were obtained. Personal history including cannabis use, last age of cannabis use, tobacco smoking, and family history (only PCa family history was available in UKB) were also obtained from UKB. The research outcomes including the diagnosis of BCa, RCC, PCa, or TCa were defined according to the International Classification of Diseases (ICD)‐10 code as C67, C64, C61, and C62. Both primary and non‐primary cancers were recruited. Age of diagnosis and follow‐up time was collected to evaluate the time effect on the outcomes.

According to the standard questionnaire of UKB (Figure S1), cannabis consumption status was classified into 5 groups: never use, 1–2 times, 3–10 times, 11–100 times, and more than 100 times. To facilitate analysis, we bisected the whole cohort into two subgroups “never use cannabis” and “ever use cannabis.” In order to distinguish previous cannabis use from current use, the age of last cannabis use was compared with the age of diagnosis or the latest follow‐up date of each individual. Therefore, the cannabis smoking status was finally classified into three groups: “never use cannabis,” “previous cannabis use” (defined as last cannabis use >2 years earlier than diagnosis or latest follow‐up date), and “current cannabis use” (defined as last cannabis use <2 years earlier than diagnosis or latest follow‐up date). The 2‐year interval to distinguish previous from current was identified by our group.

### 2.3 Statistical analysis

Baseline characteristics were illustrated by descriptive statistics. Chi‐squared test was used to compare the difference between categorical variables. The student's *t‐test* was applied to evaluate the normally distributed continuous variables while Mann–Whitney *U* test was used to evaluate non‐normally distributed continuous variables. Crude incidence ratio (IR) was calculated to illustrate the risk of diseases based on the incidences. Age‐standardized incidence ratios (SIRs) were estimated based on the crude incidence and the population age distribution of the European standard population.^15^ Univariate and multivariable Cox proportional hazards regression was further conducted to analyze the time effect (follow‐up period) on the association between cannabis use and disease risks. Recruitment age and widely acknowledged risk factors (gender and smoke status for BCa^16^; gender, smoke status, and BMI for RCC^17^; family history for PCa)^18^ were adjusted as covariates. Participants diagnosed with urological cancers before recruitment was also excluded in the Cox model with the time effect of the follow‐up period. In order to exclude the influence of other variables, Cox regression was repeated in subgroups of variables mentioned above. Among them, the cohort was stratified by recruitment age into subgroups of recruitment age ≥ 55 years and <55 years, and by BMI into subgroups of BMI ≤ 25 and >25. Analyses of PCa and TCa were conducted only in male cases.

Statistical analyses of the observational study were implemented with IBM SPSS Statistics for Windows, Version 24.0 (IBM Corp., Armonk, NY, USA). A 2‐tailed *p*‐value of 0.05 or less was considered statistically significant.

A two‐sample MR analysis was performed to evaluate the causal relationship between cannabis use (exposure) and cancer risks (outcomes). The genetic mediators in this MR analysis were obtained from a genome‐wide association study (GWAS) for lifetime cannabis use to date in European ancestry (*n* = 184,765).^19^ Single nucleotide polymorphisms (SNPs) reaching *p* < 1 × 10^−5^ (44 SNPs) were used as instrumental variables. Detailed information on MR was presented in Supplementary Materials.

Statistical analyses were implemented with IBM SPSS Statistics Version 24.0 (IBM Corp., Armonk, NY, USA) and R 4.0.3 (“TwoSampleMR” package).^20^ A 2‐tailed *p* < 0.05 was considered statistically significant.

## 3 RESULTS

**FIGURE 1:**
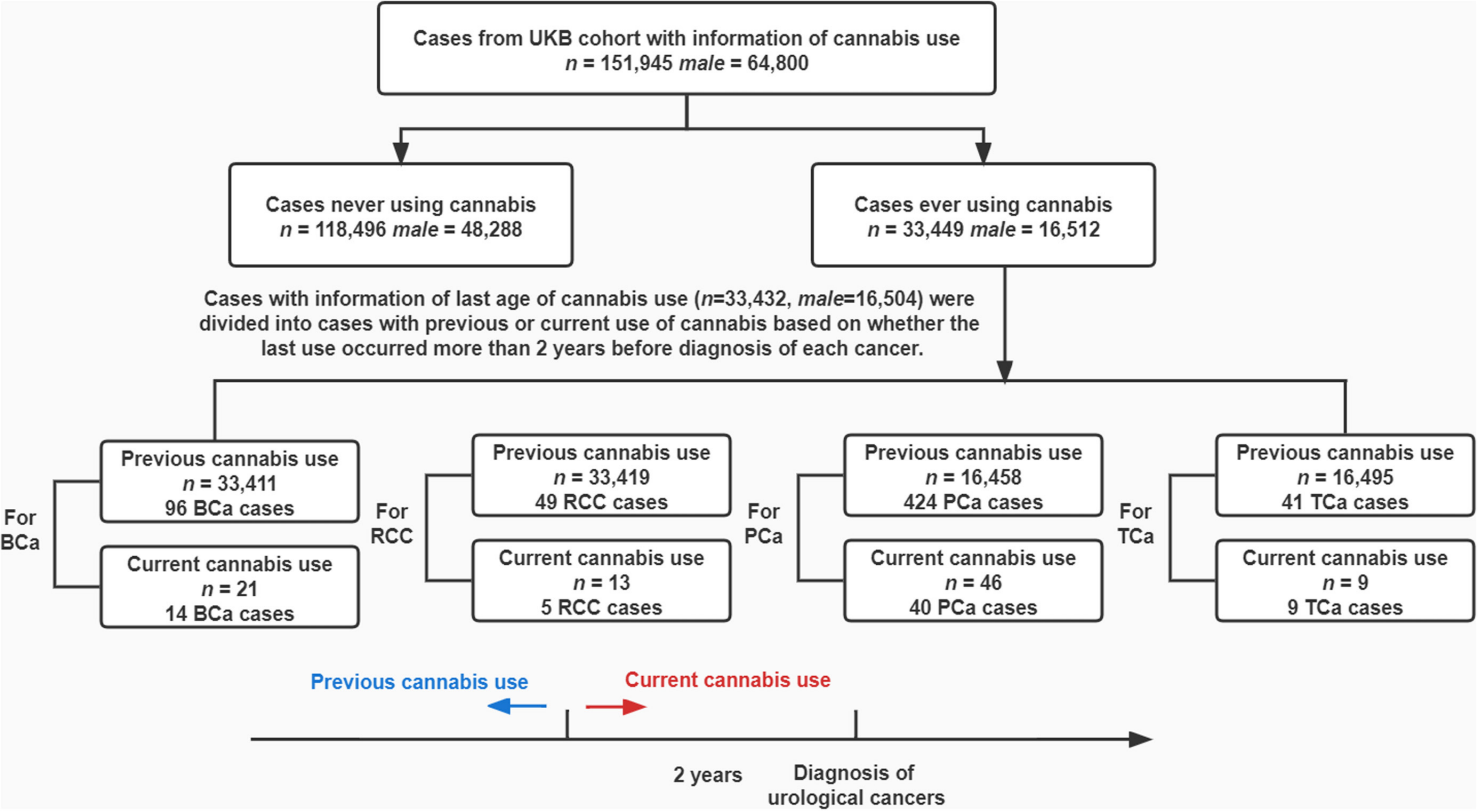
Flowchart showing the composition of the cohort.

Figure 1 shows the detailed information of the study cohort. A total of 151,945 individuals were finally included in the present study. Among them, 151,929 individuals had additional information about the age of last cannabis use. Finally, 118,496 participants were identified as “never use cannabis” and 33,449 participants were identified as “ever use cannabis”. Baseline characteristics of the study population are shown in Table 1 and Table S1. The median follow‐up time was 140.14 months (interquartile range, IQR, 132.08–148.29 months) for “never use cannabis” and 139.91 months (IQR, 131.56–147.80 months) for “ever use cannabis.”

**TABLE 1:** Characteristics of the study population from the UK Biobank Database

Characteristic	Entire cohort (*n* = 151,945)	
	Never use cannabis *n* = 118,496	Ever use cannabis *n* = 33,449
Gender, *n* (%)		
Male	48,232 (41.6)	16,498 (50.4)
Female	67,839 (58.4)	16,216 (49.6)
Overall mortality, *n* (%)	2272 (1.9)	462 (1.4)
Tabacco smoking, *n* (%)		
Never tabacco smoking (%)	76,139 (64.3)	10,421 (31.2)
Previous tabacco smoking (%)	36,386 (30.8)	17,716 (53.0)
Current tabacco smoking (%)	5796 (4.9)	5260 (15.7)
Cannabis use, *n* (%)		
Never	118,496 (100)	0 (0)
1–2 times	0 (0)	14,411 (43.1)
3–10 times	0 (0)	8330 (24.9)
11–100 times	0 (0)	6726 (20.1)
More than 100 times	0 (0)	3982 (11.9)
Recruiting age [Mean (SD)]	57.0 (7.5)	52.7 (0.3)
Follow‐up time [Mean (SD)]	140.3 (10.8)	139.9 (10.9)
BMI [Mean (SD)]	26.9 (4.6)	26.4 (4.5)
BCa, *n* (%)	618 (0.5)	110 (0.3)
BCa diagnosis age [Mean (SD)]	63.5 (7.9)	60.6 (8.3)
RCC, *n* (%)	298 (0.3)	54 (0.2)
RCC diagnosis age [Mean (SD)]	62.9 (8.1)	59.6 (8.3)
^a^TCa, *n* (%)	117 (0.2)	49 (0.3)
^a^TCa diagnosis age [Mean (SD)]	45.5 (10.8)	44.9 (9.8)
^a^PCa, *n* (%)	2471 (5.1)	451 (2.7)
^a^PCa diagnosis age [Mean (SD)]	66.0 (5.7)	63.9 (5.49)

Abbreviations: BCa, bladder cancer; BMI, body mass index; PCa, prostate cancer; RCC, renal cell carcinoma; SD, standard deviation; TCa, testicular cancer; UK, United Kingdom.

^a^ Analyses of TCa and PCa were conducted within males (*n* = 64,730) including 48,232 individuals never used cannabis and 16,498 ones ever using cannabis.

As shown in Table S2, the IRs of BCa, RCC, and PCa in “ever use cannabis” individuals were 0.3%, 0.2%, and 2.7%, respectively, significantly lower than those in “never use cannabis” participants (0.5%, 0.3%, and 5.1%, respectively, all *p* < 0.05). However, there was no difference in TCa incidence between these two groups (0.3% vs 0.2%, *p* = 0.233).

The SIRs of each disease were calculated based on the European standard population (Table S2). SIRs suggested that “ever use cannabis” individuals had significantly lower risk of BCa, RCC and PCa than “never use cannabis” individuals (SIR_BCa_ = 0.85, 95% confidence interval, 95%CI: 0.83–0.87, *p* < 0.001; SIR_RCC_ = 0.69, 95%CI: 0.67–0.71, *p* < 0.001; SIR_PCa_ = 0.51, 95% CI: 0.49–0.53, *p* < 0.001). After adjusting with age, no significant association was observed between TCa and cannabis use (SIR_TCa_ = 0.99, 95% CI: 0.96–1.02, *p* = 0.588). Multivariate Cox hazard regression showed similar trends of associations between ever use cannabis and urological cancers but not significant (HR_BCa_ = 0.86, 95% CI: 0.66–1.12, *p* = 0.292; HR_RCC_ = 0.68, 95% CI: 0.45–1.01, *p* = 0.058; HR_PCa_ = 0.89, 95% CI: 0.79–1.01, *p* = 0.065; HR_TCa_ = 0.85, 95% CI: 0.37–1.97, *p* = 0.711).

Current cannabis use had a strong association with the increased incidence of diseases (HRs > 1, all *p* < 0.001, Table S3). Previous cannabis use had a significant inverse association with RCC and PCa in terms of disease‐free survival since recruitment (HR_RCC_ = 0.61, 95% CI: 0.40–0.93; *p* = 0.021; HR_PCa_ = 0.83, 95% CI: 0.74–0.94; *p* = 0.003, Table 2) and a potential tendency to be related to lower incidence of BCa as well (HR = 0.77, 95% CI: 0.58–1.02, *p* = 0.071). However, it was not significantly associated with the risk of TCa (HR = 0.85, 95% CI: 0.37–1.97, *p* = 0.853).

**TABLE 2:** Univariable and multivariable cox regression predicting the association between previous cannabis use and urological cancers

Characteristic		n	Number of cancers	Crude HR (95%CI)	*p*‐value	Adjusted HR^b^ (95%CI)	*p*‐value
BCa	Never use cannabis	118,228	350	1.00 (ref.)	—	1.00 (ref.)	—
	Previous cannabis use	33,380	65	0.66 (0.51–0.86)	***0.002***	0.77 (0.58–1.02)	*0.071*
RCC	Never use cannabis	118,391	193	1.00 (ref.)	—	1.00 (ref.)	—
	Previous cannabis use	33,398	28	0.51 (0.35–0.76)	***0.001***	0.61 (0.40–0.93)	***0.021***
^a^PCa	Never use cannabis	47,579	1814	1.00 (ref.)	—	1.00 (ref.)	—
	Previous cannabis use	16,361	327	0.52 (0.46–0.58)	***<0.001***	0.83 (0.74–0.94)	***0.003***
^a^TCa	Never use cannabis	48,188	21	1.00 (ref.)	—	1.00 (ref.)	—
	Previous cannabis use	16,462	8	1.12 (0.49–2.52)	*0.793*	0.85 (0.37–1.97)	*0.853*

*Note*: In univariable and multivariable Cox regression, recruitment time and birth time were treated as the start point of observation.

The bold values were statistically significant.

Abbreviations: BCa, bladder cancer; CI, confidence interval; HR, hazard ratio; PCa, prostate cancer; RCC, renal cell carcinoma; TCa, testicular cancer.

^a^ Analyses of TCa and PCa were conducted within males (*n* = 64,730).

^b^ Adjusted HRs were according to the multivariable Cox hazard regression. Covariates adjusted were recruitment age and widely acknowledged risk factors (gender and smoke status for BCa; gender, smoke status, and BMI for RCC; family history for PCa).

**FIGURE 2:**
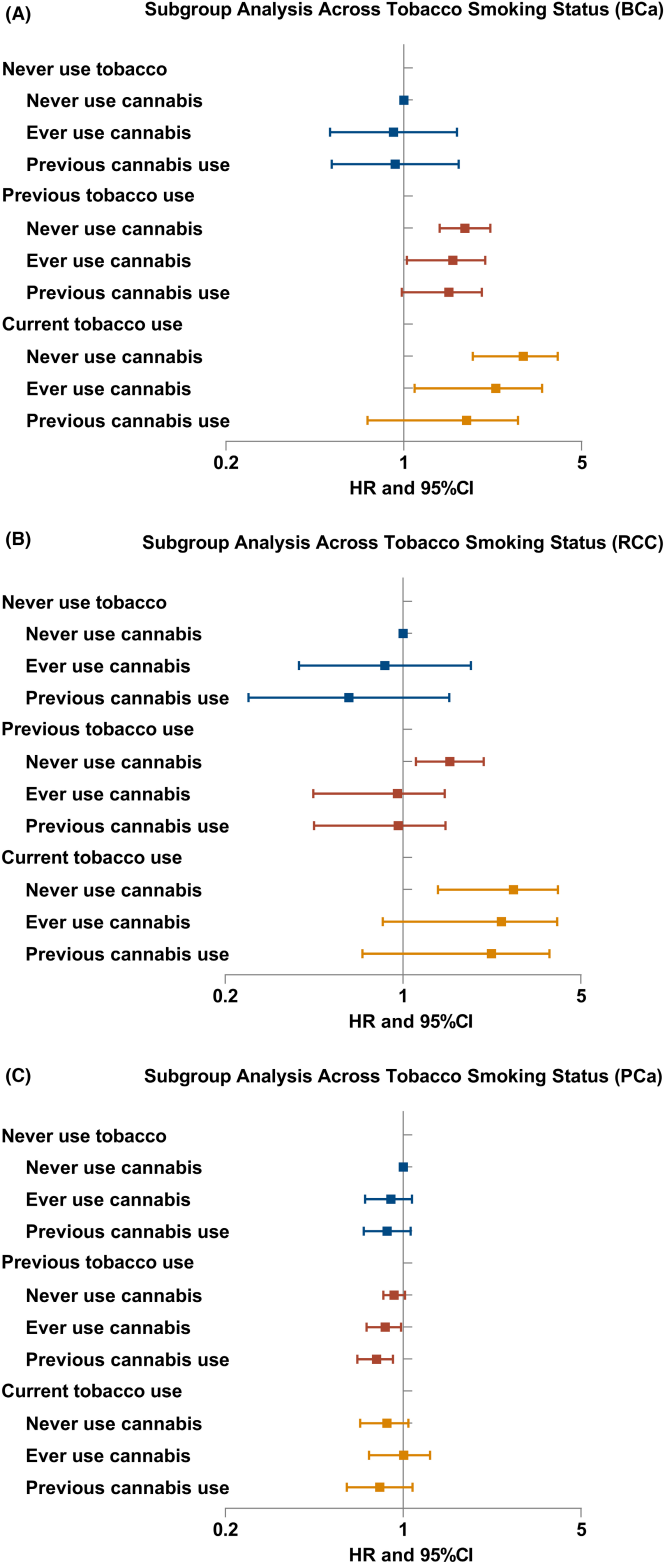
Forest plot: HRs of cannabis use in subgroups of tobacco smoking (A) for BCa; (B) for RCC; (C) for PCa according to multivariate Cox analyses. Abbreviation: BCa, bladder cancer; RCC, renal cell carcinoma; PCa, prostate cancer; HR, hazard ratio; CI, confidence interval.

**FIGURE 3:**
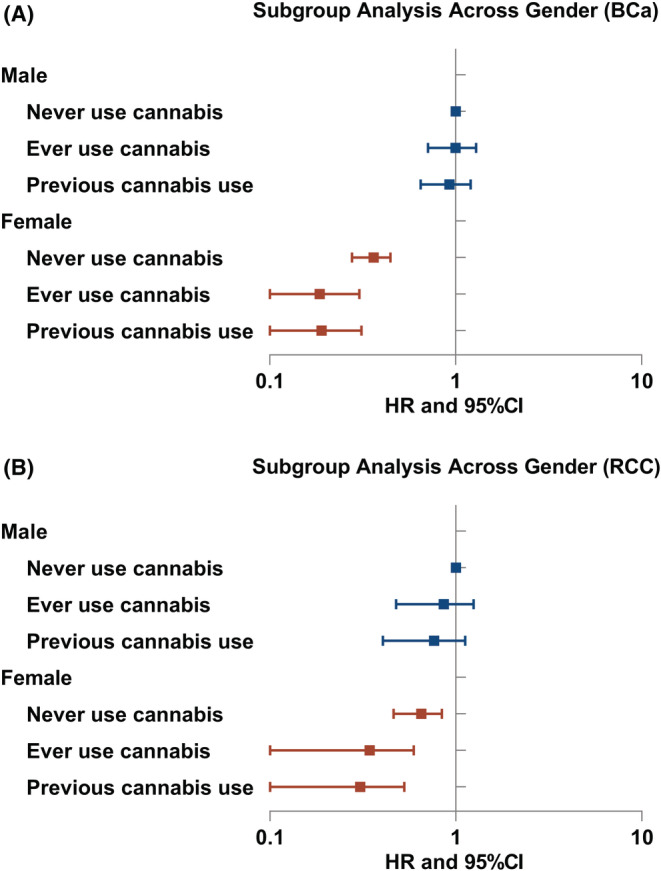
Forest plot: HRs of cannabis use in subgroups of different genders (A) for BCa; (B) for RCC. Abbreviation: BCa, bladder cancer; RCC, renal cell carcinoma; PCa, prostate cancer; HR, hazard ratio; CI, confidence interval.

Subgroup analyses were performed in different groups of gender, recruitment age, BMI, tobacco smoking status, and family history (Figure 2, 3; Figures S3–S6; Tables S4–S8). Interestingly, in most subgroups, cannabis use tended to decrease the risk of BCa, RCC, and PCa with HRs < 1. However, the association between cannabis use and TCa incidence was not significant in any subgroup analysis.

Survival analyses in subgroups using Cox regression showed that previous cannabis use was a significant protective factor for PCa in men who were with history of previous tobacco smoking (HR_PCa_ = 0.83, 95% CI: 0.70–0.99, *p* = 0.033) (Table S4; Figure 2), without a family history of PCa (HR_PCa_ = 0.81, 95% CI: 0.73–0.91, *p* < 0.001) (Table S5; Figure S3), with BMI >25 (HR_PCa_ = 0.81, 95% CI: 0.70–0.94, *p* = 0.007) (Table S6; Figure S4), and recruited both before and after 55 years old (Table S7; Figure S5).

These protective effects on BCa and RCC of cannabis use were more obvious in individuals who were currently smoking tobacco (Figure 2 and Table S4). In the subgroup analysis of gender (Figure 3 and Table S8), previous use of cannabis might reduce the risks of both RCC and BCa in females of previous cannabis use (HR_RCC_ = 0.42, 95% CI: 0.19–0.94, *p* = 0.034; HR_BCa_ = 0.43, 95% CI: 0.21–0.86, *p* = 0.018). However, the associations were not significant in men.

We then performed a series of sensitivity analyses and confirmed the results (Tables S9–S10, detail sensitivity analyses were presented in Supplementary Materials).

The results of bi‐directional two‐sample MR were shown in Table 3; Table S11 and Figures S7–S10. A causal effect of cannabis use on lower incidence of RCC was observed with IVW regression (OR = 0.72, 95% CI: 0.58–0.89, *p* < 0.001, Table 3; Figure S7). Weighted median regression and MR‐Egger presented a similar pattern as IVW regression without a noticeable horizontal pleiotropy, indicating no confounder in this causal relationship (Table 3).

**TABLE 3:** Results of the two‐sample Mendelian randomization analysis between lifetime cannabis use and BCa, RCC, and PCa including results of sensitivity analyses (significance threshold of SNPs *p* < 1e‐05)

Algorithm	Cannabis – BCa (44 SNPs)		Cannabis – RCC (44 SNPs)		Cannabis – PCa (44 SNPs)	
	OR (95%CI)	*p*‐value	OR (95%CI)	*p*‐value	OR (95%CI)	*p*‐value
IVW (RE)	0.90 (0.77–1.06)	*0.21*	0.72 (0.58–0.89)	***2.44e‐03***	1.06 (0.96–1.17)	*0.22*
IVW (FE)	0.90 (0.77–1.06)	*0.21*	0.72 (0.58–0.89)	***2.44e‐03***	1.06 (0.97–1.16)	*0.17*
Weighted median	0.91 (0.71–1.16)	*0.44*	0.81 (0.60–1.11)	*0.19*	0.98 (0.86–1.11)	*0.73*
MR Egger	1.11 (0.74–1.67)	*0.60*	0.87 (0.51–1.49)	*0.61*	0.88 (0.69–1.12)	*0.31*
MR‐Egger intercept	—	*0.27*	—	*0.46*	—	*0.46*
Heterogeneity test	—	*0.61*	—	*0.65*	—	*0.65*
Outlier‐corrected effect	N/A	N/A	N/A	N/A	N/A	N/A

*Note*: Significant results (*p* < 0.05; tested two‐sided) are shown in bold. Odds ratios represent the odds of urological cancers (BCa, RCC, and PCa) for lifetime cannabis users versus non‐users (when cannabis is the exposure) (OR).

Abbreviations: BCa, bladder cancer; CI, Confidence interval; FE, Fixed effect; IVW, Inverse Variance Weighted regression analysis; OR, Odds ratios; PCa, prostate cancer; RCC, renal cell carcinoma; RE, Random effect.

## 4 DISCUSSION

In the current study, we investigated the association between the use of cannabis and the risk of urological cancers. We observed that: (1) previous use of cannabis was a significant inverse association with both RCC and PCa; (2) cannabis use was associated with the lower risk of BCa in the point estimates; (3) the protective effect of cannabis on RCC and BCa was significant for females but not for males; (4) cannabis use had a causal effect on lower incidence of RCC.

It should be noted that the current cannabis use was significantly associated with an increased risk of urological cancers. Some of the current use probably resulted from the diagnosis of urological cancers (medical consumption). Therefore, it was cursory and inappropriate to interpret that cannabis was a risk factor. Due to the limited information in the UKB database, we were not able to distinguish cannabis use because of cancers from the current use of cannabis. Moreover, the number of cases in this group relatively small (21 BCa, 13 RCC, 47 PCa cases, and 9 TCa, respectively), which was consisted of diagnosed cases and lost follow‐up cases because of death. Therefore, the statistical power of analyses in this subgroup was limited and it was difficult to achieve any confident interpretation. Additionally, a series of subgroup analyses were performed in this study. Due to the effect of multiple tests, the results of subgroup analyses were treated prudently with a higher standard for judging the significance. However, because of the limited sample size and number of events in each subgroup, most results did not achieve significance. Tendency of the results was focused.

With the increasing social acceptance of cannabis use, several studies focused on the relationship between cannabis use and the incidence of cancers, including lung cancer, neck cancer, etc.^21^, ^22^ To date, there was no conclusive evidence available that cannabis use might affect the incidence of lung cancer and other cancers.^21^, ^22^, ^23^ Similarly, very few studies explored cannabis use and urological cancers.^7^, ^22^ Studies concluded that current strong cannabis use was a risk factor of testicular germ cell tumors.^22^ However, we were not able to observe a statistical significance between cannabis use and TCa in the present study due to the relatively small number of TCa and lack of pathologic information in the UKB. Two large‐scale cohorts indicated contradictory conclusions on the association between cannabis and BCa. A matched case–control study with only ~150 samples suggested that cannabis use might be a risk factor for BCa.^9^ The study design might bring inevitable selection bias. Another prospective study with 84,170 participants from the California Men's Health Study (CMHS) cohort indicated that cannabis use could reduce the 45% incidence of BCa.^10^ However, it is undeniable that the gender limitation of the study cohort reduced the generalizability of the conclusion. The only study about the relationship between cannabis use and PCa was published in 1997 by Sidney et al.. This retrospective study showed that ever‐use of cannabis but not tobacco was associated with an increased risk of PCa.^24^ There was no reported study regarding the effect of cannabis on RCC. Therefore, our study might be the most comprehensive study that attempted to reveal the role of cannabis in the incidence of urological cancers to date in a population cohort.

Several functional studies also investigated the relationship between cannabinoids and urological cancers. Briefly, cannabinoids might have the potential anticancer capacity.^25^, ^26^ Overexpression of CB1 or CB2 (cannabinoid receptors) was observed on RCC, PCa, and BCa cells,^27^, ^28^ which implied the potential carcinogenesis of urological cancers.^29^ On the contrary, cannabinoid agonists induced apoptosis of PCa cells and reduced the size of tumors.^12^, ^13^ Additionally, several in vivo and in vitro studies revealed the crucial role of cannabinoid receptors in the anti‐proliferation of BCa cells.^27^, ^30^

The gender‐specific protective role of cannabis in RCC and BCa was observed in the present study (only in females but not in males). Cooper et al. reviewed the observations of sex‐dependent effects of cannabis and cannabinoids in the processes of studies and clinical practique.^31^ Sex hormonal mechanism was considered as a potential reason.^32^ For example, both testosterone and estradiol were demonstrated to be able to modulate the sensitivity to the effect of THC.^33^, ^34^ In addition, the cannabinoid metabolism and receptors might function differently in males and females.^35^, ^36^ Further studies are necessary to further illustrate the unrevealed mechanism.

Several limitations should be noted. First, we were not able to interpret the results in individuals with the current use of cannabis. As mentioned, it was due to the relatively small number of cancers in this group and the lack of information about the purpose of cannabis consumption (medical or recreational) in UKB. Further study should be conducted to answer the questions. Second, all information about cannabis use was based on the questionnaire, which would cause recall bias. However, the quality control process was built in the initial design of the UKB questionnaire,^14^ which ensured the reliability of the data. Third, it was difficult to clarify occasional cannabis use. Although we conducted a sensitivity analysis to exclude its influence as much as possible, the effect of occasional and regular use remained to be explored. Fourth, partial sample overlapping is unavoidable when obtaining adequate SNPs related to cannabis lifetime use for subsequent analysis, which might bring selection bias and should be further interpreted more carefully.

## 5 CONCLUSION

Previous cannabis use correlated with a lower risk of BCa, PCa, and RCC. Especially, cannabis use had a causal effect on a lower incidence of RCC. The inverse association between cannabis and either RCC or BCa was significant in females but not in males. However, no specific effect of ever or previous cannabis use on the incidence of TCa was found in this study.

## AUTHOR CONTRIBUTION

Rong Na, Jingyi Huang, Da Huang, and Jonathan Olivier were responsible for the study concept and study design. Rong Na and Da Huang acquired the data. Jingyi Huang, Xiaohao Ruan, and Susan Heavey analyzed and interpreted the data. Jingyi Huang, Da Huang, Xiaohao Ruan, and Jinlun Huang drafted the manuscript. Rong Na, Jonathan Olivier, Susan Heavey, and Danfeng Xu contributed to the critical revision of the manuscript. Rong Na and Jonathan Olivier supervised the study. All authors have read and approved the final manuscript.

## FUNDING INFORMATION

This work was supported by grants from the National Natural Science Foundation of China (Grant No. 81972645) and the Shanghai Youth Talent Support Program to Prof. Rong Na, and Shanghai Sailing Program (22YF1440500) to Dr. Da Huang.

## CONFLICT OF INTEREST

The authors declare to have no conflict of interest.

## ETHICS STATEMENT

This study was conducted in the cohort from the UK Biobank, which is a public database. The project of the present study was approved by the UK biobank so ethics approval was not applicable.

## Supporting information


Appendix S1

Table S12

Table S13

## Data Availability

We thank the UK Biobank for access to the data (project number: 66813). All data used in this research are publicly available to qualified researchers on application to the UK Biobank (www.ukbiobank.ac.uk).
