# Supplementary material for: Association between cannabis use with urological cancers: A population‐based cohort study and a mendelian randomization study in the UK biobank
Source: Cancer Med. 2022 Aug 17;12(3):3468–76. doi: 10.1002/cam4.5132 (PMC9939109; doi:10.1002/cam4.5132)
Supplement: Supplementary file 1 — Appendix S1 [file CAM4-12-3468-s001.docx]

**Supplementary Methods**

**Sensitivity Analyses**

Sensitivity analyses were performed.

First, so as to exclude medical use of cannabis because of the disease and clarify the effect of previous use, we focused on the association between use of cannabis before recruitment to UKB cohort and incidence of urological cancers after recruitment. Cases were excluded whose last use of cannabis was after recruitment.

Second, it was hard to identify occasional use and regular use of cannabis. In order to exclude the influence of occasional use and make the result reliable, cases with a few times of cannabis use were categorized into group of never use cannabis. Two different grouping patterns were used: 1) 0-2 times cannabis use 2) 0-10 times cannabis use was identified as never use cannabis.

**Two-sample Mendelian randomization**

We conducted two-sample MR,^1^ which uses genetic variants associated with the exposure as instrumental variables, to explore whether there existed any causal relationship between cannabis use and urological cancers. Bi-directional effects were tested in the present study. Due to the limited size of cases with TCa in the cohort, MR about TCa was not performed. The process of MR was presented by the flowchart in **Figure S2**.

For cannabis use as an exposure, instrumental variable selection was based on the largest genome-wide association study (GWAS) for lifetime cannabis use to date in European ancestry (n=184,765), whose data were from the International Cannabis Consortium (ICC) study (n=35,297), 23andMe (n=22,683) and UK-Biobank (n=126,785).^2^ Single nucleotide polymorphisms (SNPs) that met two different criteria were proposed as instrumental variables: 1) the genome-wide statistical significance threshold *p*<5e-08 (8 SNPs); 2) the more lenient significance threshold *p*<1e-05 (44 SNPs). All SNPs satisfied the criteria: r2 < 0.05 and linkage disequilibrium window of 10,000 kb. After harmonization, two instruments were consistent with 8 SNPs (*p*<5e-08) and 44 SNPs (*p*<1e-05) relatively. For urological cancer, genetic variants were obtained from published summary statistics data in autosome in European ancestry. SNPs which met the significance threshold *p*<5e-08 were included and underwent harmonization to work as instrumental variables for BCA (13 SNPs), RCC (14 SNPs) and PCA (132 SNPs). Detailed information for all instrumental variables were noted in **Table S12-13**. The information for outcome were based on UKB cohort.

The inverse variant-weighted (IVW) method with either fixed-effects or random-effects^3^ was applied to assess the association between cannabis use and urological cancers. Cochrane’s Q statistic was used to measure heterogeneity among instrumental variables so as to determine IVW’s effect type. Several robust methods and sensitivity analyses were also conducted and described as below. In order to detect and adjust for horizontal pleiotropy, MR-Egger^4^ and MR Pleiotropy RESidual Sum and Outlier (MR-PRESSO) methods^5^ were performed. Additionally, the weighted median^6^ approach and the leave-one-out analyses verify the stability and reliability of the results.

**Reference**

1. Burgess S, Davey Smith G, Davies NM, et al. Guidelines for performing Mendelian randomization investigations. Wellcome Open Res. 2019;4:186; doi: 10.12688/wellcomeopenres.15555.2.

2. Pasman JA, Verweij KJH, Gerring Z, et al. GWAS of lifetime cannabis use reveals new risk loci, genetic overlap with psychiatric traits, and a causal influence of schizophrenia. Nature neuroscience. 2018;21(9):1161-1170; doi: 10.1038/s41593-018-0206-1.

3. Burgess S, Butterworth A, Thompson SG. Mendelian randomization analysis with multiple genetic variants using summarized data. Genetic epidemiology. 2013;37(7):658-665; doi: 10.1002/gepi.21758.

4. Bowden J, Davey Smith G, Burgess S. Mendelian randomization with invalid instruments: effect estimation and bias detection through Egger regression. International journal of epidemiology. 2015;44(2):512-525; doi: 10.1093/ije/dyv080.

5. Verbanck M, Chen CY, Neale B, et al. Detection of widespread horizontal pleiotropy in causal relationships inferred from Mendelian randomization between complex traits and diseases. Nature genetics. 2018;50(5):693-698; doi: 10.1038/s41588-018-0099-7.

6. Bowden J, Davey Smith G, Haycock PC, et al. Consistent Estimation in Mendelian Randomization with Some Invalid Instruments Using a Weighted Median Estimator. Genetic epidemiology. 2016;40(4):304-314; doi: 10.1002/gepi.21965.

| **Table S1. Characteristics of the Study Population from UK Biobank Database** | | | | |
| --- | --- | --- | --- | --- |
| **Characteristic** | | **Entire Cohort (n = 151,945)** | | |
|  |  | **Never Use Cannabis n=118,496** | **Ever Use Cannabis n=33,449** | ***P* value** |
| **Gender, n (%)** | |  |  | ***<0.001*** |
|  | **Male** | 48,232 (41.6) | 16,498 (50.4) |  |
|  | **Female** | 67,839 (58.4) | 16,216 (49.6) |  |
| **Overall Mortality, n (%)** | | 2,272 (1.9) | 462 (1.4) | ***<0.001*** |
| **Tabacco Smoking, n (%)** | |  |  | ***<0.001*** |
|  | **Never Tabacco Smoking (%)** | 76,139 (64.3) | 10,421 (31.2) |  |
|  | **Previous Tabacco Smoking (%)** | 36,386 (30.8) | 17,716 (53.0) |  |
|  | **Current Tabacco Smoking (%)** | 5,796 (4.9) | 5,260 (15.7) |  |
| **Recruiting Age [Mean (SD)]** | | 57.0 (7.5) | 52.7 (0.3) | ***<0.001*** |
| **Follow-up time [Mean (SD)]** | | 140.3 (10.8) | 139.9 (10.9) | ***<0.001*** |
| **BMI [Mean (SD)]** | | 26.9 (4.6) | 26.4 (4.5) | ***<0.001*** |
| **BCa, n (%)** | | 618 (0.5) | 110 (0.3) | ***<0.001*** |
| **BCa Diagnosis Age [Mean (SD)]** | | 63.5 (7.9) | 60.6 (8.3) | ***<0.001*** |
| **RCC, n (%)** | | 298 (0.3) | 54 (0.2) | ***0.002*** |
| **RCC Diagnosis Age [Mean (SD)]** | | 62.9 (8.1) | 59.6 (8.3) | ***0.009*** |
| **^a^TCa, n (%)** | | 117 (0.2) | 49 (0.3) | *0.233* |
| **^a^TCa Diagnosis Age [Mean (SD)]** | | 45.5 (10.8) | 44.9 (9.8) | *0.802* |
| **^a^PCa, n (%)** | | 2,471 (5.1) | 451 (2.7) | ***<0.001*** |
| **^a^PCa Diagnosis Age [Mean (SD)]** | | 66.0 (5.7) | 63.9(5.49) | ***<0.001*** |
| Abbreviation: UK, United Kingdom; BMI, body mass index; BCa, bladder cancer; RCC, renal cell carcinoma; PCa, prostate cancer; TCa, testicular cancer; SD, standard deviation ^a^ Analyses of TCa and PCa were conducted within males (n=64,730) including 48,232 individuals never using cannabis and 16,498 ones ever using cannabis. | | | | |

| **Table S2. Association of Cannabis Use with Incidence of Urological Cancer** | | | | | | | | |
| --- | --- | --- | --- | --- | --- | --- | --- | --- |
| **Urological Cancer** | **Never Use Cannabis** | **Ever Use Cannabis** | **Crude IR (95% CI)** | ***P value*** | **Age-SIR (95% CI)** | ***P value*** | **Multivariable adjusted HR**^b^ **(95% CI)** | ***P value*** |
| **BCa (%)** | 618 (0.5) | 110 (0.3) | 0.63 (0.51-0.77) | ***<0.001*** | 0.85 (0.83-0.87) | ***<0.001*** | 0.86 (0.66-1.12) | *0.292* |
| **RCC (%)** | 298 (0.3) | 54 (0.2) | 0.64 (0.48-0.86) | ***0.002*** | 0.69 (0.67-0.71) | ***<0.001*** | 0.68 (0.45-1.01) | *0.058* |
| **^a^PCa (%)** | 2,471 (5.1) | 451 (2.7) | 0.52 (0.47-0.58) | ***<0.001*** | 0.51 (0.49-0.53) | ***<0.001*** | 0.89 (0.79-1.01) | *0.065* |
| **^a^TCa (%)** | 117 (0.2) | 49 (0.3) | 1.23 (0.88-1.72) | *0.233* | 0.99 (0.96-1.02) | *0.588* | 0.85 (0.37-1.97) | *0.711* |

Abbreviation: SIR, standardized incidence ratio; IR, incidence ratio; HR, hazard ratio; CI, confidence interval; BCa, bladder cancer; RCC, renal cell carcinoma; PCa, prostate cancer; TCa, testicular cancer
^a^ Analyses of TCa and PCa were conducted within males (n=64,730) including 48,232 individuals never using cannabis and 16,498 ones ever using cannabis.

^b^Multivariable adjusted HRs were according to the multivariable Cox hazard regression. Covariates adjusted were recruitment age and widely acknowledged risk factors (gender and smoke status for BCa; gender, smoke status and BMI for RCC; family history for PCa)

| **Table S3. Univariable and Multivariable Cox Regression Predicting the Association Between Current Cannabis Use and Urological Cancers** | | | | | | | |
| --- | --- | --- | --- | --- | --- | --- | --- |
| **Characteristic** | | n | Number of cancers | Crude HR (95%CI) | *p* value | Adjusted HR* (95%CI) | *p* value |
| **BCa** | Never Use Cannabis | 118,228 | 350 | 1.00 (ref.) | *-* | 1.00 (ref.) | *-* |
|  | Current Cannabis Use | 13 | 6 | 211.59 (94.40-474.26) | ***< 0.001*** | 112.22 (49.74-253.22) | ***< 0.001*** |
| **RCC** | Never Use Cannabis | 118,391 | 193 | 1.00 (ref.) | *-* | 1.00 (ref.) | *-* |
|  | Current Cannabis Use | 11 | 3 | 200.43 (64.06-627.04) | ***< 0.001*** | 129.50 (40.97-409.34) | ***< 0.001*** |
| **^a^PCa** | Never Use Cannabis | 47,579 | 1,814 | 1.00 (ref.) | *-* | 1.00 (ref.) | *-* |
|  | Current Cannabis Use | 33 | 27 | 33.62 (22.98-49.19) | ***< 0.001*** | 43.31 (29.59-63.38) | ***< 0.001*** |
| **^a^TCa** | Never Use Cannabis | 48,188 | 21 | 1.00 (ref.) | *-* | 1.00 (ref.) | *-* |
|  | Current Cannabis Use | 0 | 0 | *-* | *-* | *-* | *-* |

Abbreviation: HR, hazard ratio; CI, confidence interval; BCa, bladder cancer; RCC, renal cell carcinoma; PCa, prostate cancer; TCa, testicular cancer
In univariable and multivariable Cox regression, recruitment time and birth time were treated as the start point of observation.
*Adjusted HRs were according to the multivariable Cox hazard regression. Covariates adjusted were recruitment age and widely acknowledged risk factors (gender and smoke status for BCa; gender, smoke status and BMI for RCC; family history for PCa).

^a^ Analyses of TCa and PCa were conducted within males (n=64,730).

| **Table S4. Multivariable Cox Regression Predicting Incidence of BCa, RCC, PCa and TCa in Subgroups of Tobacco Smoking Status** | | | | | | | | | | | | | | | |
| --- | --- | --- | --- | --- | --- | --- | --- | --- | --- | --- | --- | --- | --- | --- | --- |
| **Characteristic** | | Never Use Tobacco | | | |  | Previous Tobacco Use | | | |  | Current Tobacco Use | | | |
|  |  | N | n | Adjusted HR^b^ (95%CI) | *p* value |  | N | n | Adjusted HR^b^ (95%CI) | *p* value |  | N | n | Adjusted HR^b^ (95%CI) | *p* value |
| **RCC** | Never Use Cannabis | 76,084 | 84 | 1.00 (ref.) | *-* |  | 36,343 | 91 | 1.00 (ref.) | *-* |  | 5,789 | 18 | 1.00 (ref.) | *-* |
|  | Previous Cannabis Use | 10,412 | 5 | 0.63  (0.25-1.56) | *0.313* |  | 17,686 | 15 | 0.54  (0.30-0.95) | *0.032* |  | 5,248 | 8 | 0.75  (0.31-1.79) | *0.510* |
| **BCa** | Never Use Cannabis | 76,017 | 142 | 1.00 (ref.) | *-* |  | 36,262 | 171 | 1.00 (ref.) | *-* |  | 5,775 | 36 | 1.00 (ref.) | *-* |
|  | Previous Cannabis Use | 10,409 | 13 | 0.94  (0.53-1.68) | *0.84* |  | 17,679 | 43 | 0.83  (0.58-1.20) | *0.322* |  | 5,240 | 11 | 0.49  (0.24-1.00) | *0.049* |
| ^a^**PCa** | Never Use Cannabis | 28,162 | 973 | 1.00 (ref.) | *-* |  | 16,599 | 746 | 1.00 (ref.) | *-* |  | 2,752 | 91 | 1.00 (ref.) | *-* |
|  | Previous Cannabis Use | 5,212 | 96 | 0.86  (0.70-1.07) | *0.173* |  | 8,358 | 182 | 0.83  (0.70-0.99) | *0.033* |  | 2,771 | 49 | 1.02  (0.70-1.50) | *0.903* |
| ^a^**TCa** | Never Use Cannabis | 2,8477 | 10 | 1.00 (ref.) | *-* |  | 16,859 | 10 | 1.00 (ref.) | *-* |  | 2,784 | 1 | 1.00 (ref.) | *-* |
|  | Previous Cannabis Use | 5,235 | 3 | 1.22  (0.33-4.56) | *0.765* |  | 8,418 | 3 | 0.35  (0.09-1.38) | *0.133* |  | 2,789 | 2 | 2.00  (0.15-26.05) | *0.598* |

Abbreviation: HR, hazard ratio; CI, confidence interval; BCa, bladder cancer; RCC, renal cell carcinoma; PCa, prostate cancer; TCa, testicular cancer; N, number of cases; n, number of events (cancers)
In this multivariable Cox regression, recruitment time and birth time were treated as the start point of observation.
^a^ Analyses of TCa and PCa were conducted within males.

^b^Adjusted HRs were according to the multivariable Cox hazard regression. Covariates adjusted were recruitment age and widely acknowledged risk factors (gender for BCa; gender, BMI for RCC; family history for PCa).

| **Table S5. Multivariable Cox Regression Predicting Incidence of PCa in Subgroups of PCa Family History** | | | | | | | | | | |
| --- | --- | --- | --- | --- | --- | --- | --- | --- | --- | --- |
| **Characteristic** | | Without Family History | | | |  | With Family History | | | |
|  |  | N | n | Adjusted HR^b^ (95%CI) | *p* value |  | N | n | Adjusted HR^b^ (95%CI) | *p* value |
| ^a^**PCa** | Never Use Cannabis | 46,218 | 1,699 | 1.00 (ref.) | *-* |  | 798 | 91 | 1.00 (ref.) | *-* |
|  | Previous Cannabis Use | 15,921 | 302 | 0.82 (0.72-0.93) | ***0.002*** |  | 177 | 18 | 1.07 (0.64-1.80) | *0.795* |

Abbreviation: HR, hazard ratio; CI, confidence interval; PCa, prostate cancer; N, number of cases; n, number of events (cancers)
In this multivariable Cox regression, recruitment time and birth time were treated as the start point of observation.
^a^ Analyses of TCa and PCa were conducted within males.

^b^Adjusted HRs were according to the multivariable Cox hazard regression. Covariates adjusted were recruitment age.

| **Table S6. Multivariable Cox Regression Predicting Incidence of RCC, BCa, PCa and TCa in Subgroups of BMI** | | | | | | | | | | |  |
| --- | --- | --- | --- | --- | --- | --- | --- | --- | --- | --- | --- |
| **Characteristic** | | BMI ≤ 25 | | | |  | BMI > 25 | | | | |
|  |  | N | n | Adjusted HR^b^ (95%CI) | *p* value |  | N | n | Adjusted HR^b^ (95%CI) | *p* value | |
| **RCC** | Never Use Cannabis | 44,763 | 42 | 1.00 (ref.) | *-* |  | 73,390 | 151 | 1.00 (ref.) | *-* | |
|  | Previous Cannabis Use | 14,091 | 6 | 0.50 (0.20-1.24) | *0.134* |  | 19,253 | 22 | 0.66 (0.40-1.05) | *0.079* | |
| **BCa** | Never Use Cannabis | 44,710 | 86 | 161.00 (ref.) | *-* |  | 73,280 | 261 | 1.00 (ref.) | *-* | |
|  | Previous Cannabis Use | 14,079 | 16 | 0.67 (0.38-1.18) | *0.164* |  | 19,247 | 49 | 0.84 (0.61-1.17) | *0.306* | |
| ^a^**PCa** | Never Use Cannabis | 13,478 | 509 | 1.00 (ref.) | *-* |  | 34,018 | 1,303 | 1.00 (ref.) | *-* | |
|  | Previous Cannabis Use | 5,324 | 109 | 0.87 (0.70-1.08) | *0.206* |  | 11,015 | 217 | 0.81 (0.70-0.94) | *0.007* | |
| ^a^**TCa** | Never Use Cannabis | 13,637 | 7 | 1.00 (ref.) | *-* |  | 34,468 | 14 | 1.00 (ref.) | *-* | |
|  | Previous Cannabis Use | 5,376 | 1 | 0.34 (0.04-2.89) | *0.324* |  | 11,064 | 7 | 1.10 (0.43-2.81) | *0.837* | |

Abbreviation: HR, hazard ratio; CI, confidence interval; BCa, bladder cancer; RCC, renal cell carcinoma; PCa, prostate cancer; TCa, testicular cancer; N, number of cases; n, number of events (cancers)
In this multivariable Cox regression, recruitment time and birth time were treated as the start point of observation.
^a^ Analyses of TCa and PCa were conducted within males.

^b^Adjusted HRs were according to the multivariable Cox hazard regression. Covariates adjusted were recruitment age and widely acknowledged risk factors (gender for BCa; gender, BMI for RCC; family history for PCa).

| **Table S7. Multivariable Cox Regression Predicting Incidence of RCC, BCa, PCa and TCa in Subgroups of Age** | | | | | | | | | | |  |
| --- | --- | --- | --- | --- | --- | --- | --- | --- | --- | --- | --- |
| **Characteristic** | | Recruitment age < 55yrs | | | |  | Recruitment age ≥ 55yrs | | | | |
|  |  | N | n | Adjusted HR^b^ (95%CI) | *p* value |  | N | n | Adjusted HR^b^ (95%CI) | *p* value | |
| **RCC** | Never Use Cannabis | 41,494 | 24 | 1.00 (ref.) | *-* |  | 76,897 | 169 | 1.00 (ref.) | *-* | |
|  | Previous Cannabis Use | 19,068 | 4 | 0.39 (0.13-1.19) | *0.097* |  | 14,330 | 24 | 0.70 (0.45-1.09) | *0.117* | |
| **BCa** | Never Use Cannabis | 41,489 | 34 | 161.00 (ref.) | *-* |  | 76,739 | 316 | 1.00 (ref.) | *-* | |
|  | Previous Cannabis Use | 19,064 | 11 | 0.56 (0.27-1.18) | *0.127* |  | 14,316 | 54 | 0.84 (0.62-1.13) | *0.251* | |
| ^a^**PCa** | Never Use Cannabis | 14,629 | 150 | 1.00 (ref.) | *-* |  | 32,950 | 1,664 | 1.00 (ref.) | *-* | |
|  | Previous Cannabis Use | 8,871 | 49 | 0.63 (0.45-0.87) | *0.005* |  | 7,490 | 278 | 0.85 (0.75-0.97) | *0.017* | |
| ^a^**TCa** | Never Use Cannabis | 14,602 | 10 | 1.00 (ref.) | *-* |  | 33,586 | 11 | 1.00 (ref.) | *-* | |
|  | Previous Cannabis Use | 8,868 | 5 | 0.75 (0.25-2.20) | *0.596* |  | 7,594 | 3 | 1.04 (0.28-3.84) | *0.952* | |

Abbreviation: HR, hazard ratio; CI, confidence interval; BCa, bladder cancer; RCC, renal cell carcinoma; PCa, prostate cancer; TCa, testicular cancer; N, number of cases; n, number of events (cancers)
In this multivariable Cox regression, recruitment time and birth time were treated as the start point of observation.
^a^ Analyses of TCa and PCa were conducted within males.

^b^Adjusted HRs were according to the multivariable Cox hazard regression. Covariates adjusted were recruitment age and widely acknowledged risk factors (gender for BCa; gender, BMI for RCC; family history for PCa).

| **Table S8. Multivariable Cox Regression Predicting Incidence of RCC and BCa in Subgroups of Gender** | | | | | | | | | | |  |
| --- | --- | --- | --- | --- | --- | --- | --- | --- | --- | --- | --- |
| **Characteristic** | | Male | | | |  | Female | | | | |
|  |  | N | n | Adjusted HR^b^ (95%CI) | *p* value |  | N | n | Adjusted HR^b^ (95%CI) | *p* value | |
| **RCC** | Never Use Cannabis | 48,232 | 110 | 1.00 (ref.) | *-* |  | 67,793 | 79 | 1.00 (ref.) | *-* | |
|  | Previous Cannabis Use | 16,477 | 21 | 0.90 (0.66-1.23) | *0.520* |  | 16,199 | 7 | 0.43 (0.21-0.86) | *0.018* | |
| **BCa** | Never Use Cannabis | 48,097 | 243 | 161.00 (ref.) | *-* |  | 67,764 | 98 | 1.00 (ref.) | *-* | |
|  | Previous Cannabis Use | 16,465 | 56 | 0.72 (0.44-1.18) | *0.192* |  | 16,194 | 9 | 0.42 (0.19-0.94) | *0.034* | |

Abbreviation: HR, hazard ratio; CI, confidence interval; BCa, bladder cancer; RCC, renal cell carcinoma; N, number of cases; n, number of events (cancers)
In this multivariable Cox regression, recruitment time and birth time were treated as the start point of observation.

^b^Adjusted HRs were according to the multivariable Cox hazard regression. Covariates adjusted were recruitment age and widely acknowledged risk factors (gender for BCa; gender, BMI for RCC).

| **Table S9.**  **Univariable and Multivariable Cox Regression Predicting Incidence of BCa, RCC, PCa and TCa in Cases Whose Last Cannabis was Before Recruitment** | | | | | | | |
| --- | --- | --- | --- | --- | --- | --- | --- |
| **Characteristic** | | N | n | Crude HR (95%CI) | *p* value | Adjusted HR^b^ (95%CI) | *p* value |
| **BCa** | Never Use Cannabis | 118,228 | 350 | 1.00 (ref.) | *-* | 1.00 (ref.) | *-* |
| **n=147,793** | Previous Cannabis Use | 29,565 | 62 | 0.71 (0.54-0.93) | ***0.012*** | 0.85 (0.64-1.12) | *0.245* |
| **RCC** | Never Use Cannabis | 118,391 | 193 | 1.00 (ref.) | *-* | 1.00 (ref.) | *-* |
| **n=147,968** | Previous Cannabis Use | 29,577 | 27 | 0.56 (0.37-0.84) | ***0.005*** | 0.67 (0.44-1.03) | *0.067* |
| **^a^PCa** | Never Use Cannabis | 47,579 | 1,814 | 1.00 (ref.) | *-* | 1.00 (ref.) | *-* |
| **n=61,801** | Previous Cannabis Use | 14,222 | 314 | 0.57 (0.51-0.65) | ***0.000*** | 0.89 (0.79-1.01) | *0.073* |
| **^a^TCa** | Never Use Cannabis | 48,188 | 21 | 1.00 (ref.) | *-* | 1.00 (ref.) | *-* |
| **n=62,472** | Previous Cannabis Use | 14,284 | 8 | 1.29 (0.57-2.90) | *0.546* | 1.00 (0.43-2.30) | *0.996* |

Abbreviation: HR, incidence ratio; CI, confidence interval; BCa, bladder cancer; RCC, renal cell carcinoma; PCa, prostate cancer; TCa, testicular cancer; N, number of cases; n, number of events (cancers)
In univariable and multivariable Cox regression, recruitment time and birth time were treated as the start point of observation.
^a^ Analyses of TCa and PCa were conducted within males.

^b^Adjusted HRs were according to the multivariable Cox hazard regression. Covariates adjusted were recruitment age and widely acknowledged risk factors (gender and smoke status for BCa; gender, smoke status and BMI for RCC; family history for PCa)

| **Table S10. Sensitivity Analysis by Taking Occasional Cannabis Use as Never Use** | | | | | | | | | | |
| --- | --- | --- | --- | --- | --- | --- | --- | --- | --- | --- |
| **Characteristic** | | ^b^Cannabis use 0-2 times Versus > 2 times | | | |  | ^c^Cannabis use 0-10 times Versus > 10 times | | | |
|  |  | N | n | Adjusted HR^d^ (95%CI) | *p* value |  | N | n | Adjusted HR^d^ (95%CI) | *p* value |
| **BCa** | Never Use Cannabis | 129,719 | 382 | 1.00 (ref.) | - |  | 137,867 | 398 | 1.00 (ref.) | - |
|  | Previous Cannabis Use | 18,577 | 33 | 0.71 (0.49-1.03) | *0.075* |  | 10,429 | 17 | 0.66 (0.40-1.09) | *0.100* |
|  | Current Cannabis Use | 13 | 6 | 114.06 (50.61-255.04) | *< 0.001* |  | 13 | 6 | 115.64 (51.35-260.44) | *< 0.001* |
| **RCC** | Never Use Cannabis | 129,669 | 206 | 1.00 (ref.) | - |  | 137,811 | 215 | 1.00 (ref.) | - |
|  | Previous Cannabis Use | 18,559 | 15 | 0.63 (0.37-1.09) | *0.096* |  | 10,417 | 6 | 0.45 (0.20-1.03) | *0.059* |
|  | Current Cannabis Use | 11 | 3 | 136.25 (43.16-430.06) | *< 0.001* |  | 11 | 3 | 137.14 (43.49-432.51) | *< 0.001* |
| **^a^PCa** | Never Use Cannabis | 53,372 | 1,982 | 1.00 (ref.) | - |  | 53,372 | 2,061 | 1.00 (ref.) | - |
|  | Previous Cannabis Use | 9,742 | 159 | 0.75 (0.63-0.88) | *0.001* |  | 5,702 | 80 | 0.70 (0.56-0.88) | *0.002* |
|  | Current Cannabis Use | 32 | 27 | 43.53 (29.75-63.69) | *< 0.001* |  | 32 | 27 | 43.95 (30.04-64.29) | *< 0.001* |
| **^a^TCa** | Never Use Cannabis | 54,667 | 25 | 1.00 (ref.) | - |  | 58.782 | 27 | 1.00 (ref.) | - |
|  | Previous Cannabis Use | 9,983 | 4 | 0.65 (0.22-1.90) | *0.427* |  | 5,868 | 2 | 0.54 (0.13-2.32) | *0.410* |
|  | Current Cannabis Use | 0 |  | - | *-* |  | 0 |  | - | *-* |

Abbreviation: HR, hazard ratio; CI, confidence interval; BCa, bladder cancer; RCC, renal cell carcinoma; PCa, prostate cancer; TCa, testicular cancer; N, number of cases; n, number of events (cancers)
In this multivariable Cox regression, recruitment time was treated as the start point of observation.
^a^ Analyses of TCa and PCa were conducted within males.

^b^ Cases with 1-2 times cannabis use were categorized to group of never use cannabis.

^c^ Cases with 1-10 times cannabis use were categorized to group of never use cannabis.

^d^Adjusted IRs were according to the multivariable Cox hazard regression. Covariates adjusted were recruitment age and widely acknowledged risk factors (gender and smoke status for BCa; gender, smoke status and BMI for RCC; family history for PCa)

| **Table S11. Results of the Bidirectional Two-sample Mendelian Randomization Analysis Between Lifetime Cannabis Use and Urological Cancers Including Results of Sensitivity Analyses (Significance Threshold of SNPs *p* < 5e-8).** | | | | | | | | | | | | | | | | | |
| --- | --- | --- | --- | --- | --- | --- | --- | --- | --- | --- | --- | --- | --- | --- | --- | --- | --- |
| **Algorithm** | **Cannabis - BCa** | |  | **Cannabis - RCC** | |  | **Cannabis - PCa** | |  | **BCa - Cannabis** | |  | **RCC - Cannabis** | |  | **PCa - Cannabis** | |
|  | **(8 SNPs)** | |  | **(8 SNPs)** | |  | **(8 SNPs)** | |  | **(13 SNPs)** | |  | **(14 SNPs)** | |  | **(132 SNPs)** | |
|  | **OR (95%CI)** | ***p* value** |  | **OR (95%CI)** | ***p* value** |  | **OR (95%CI)** | ***p* value** |  | **OR (95%CI)** | ***p* value** |  | **OR (95%CI)** | ***p* value** |  | **OR (95%CI)** | ***p value*** |
| **IVW (RE)** | 0.96  (0.73 - 1.26) | *0.75* |  | 0.73 (0.39 - 1.37) | *0.33* |  | 1.06  (0.96 - 1.17) | *0.22* |  | 1.02  (0.99 - 1.06) | *0.23* |  | 1.03  (0.99 - 1.07) | *0.11* |  | 0.99  (0.96 - 1.02) | *0.66* |
| **IVW (FE)** | 0.96  (0.73 - 1.26) | *0.75* |  | 0.73  (0.50 - 1.06) | *0.10* |  | 1.06  (0.97 - 1.16) | *0.17* |  | 1.02  (0.99 - 1.06) | *0.23* |  | 1.03  (0.99 - 1.07) | *0.11* |  | 0.99  (0.96 - 1.02) | *0.65* |
| **Weighted median** | 0.88  (0.62 - 1.26) | *0.5* |  | 0.84  (0.51 - 1.38) | *0.49* |  | 0.98 (0.86 - 1.11) | *0.73* |  | 1.02  (0.97 - 1.08) | *0.36* |  | 1.03  (0.98 - 1.08) | *0.29* |  | 1.00  (0.95 - 1.04) | *0.84* |
| **MR Egger** | 0.91  (0.44 - 1.89) | *0.81* |  | 0.88  (0.15 - 5.31) | *0.89* |  | 0.88  (0.69 - 1.12) | *0.31* |  | 0.98  (0.88 - 1.10) | *0.74* |  | 1.07  (0.97 - 1.19) | *0.19* |  | 0.99  (0.93 - 1.05) | *0.67* |
| **MR-Egger intercept** | - | *0.89* |  | - | *0.84* |  | - | *0.46* |  | - | *0.45* |  | - | *0.41* |  | - | *0.81* |
| **Heterogeneity test** | - | *0.81* |  | - | ***0.01*** |  | - | *0.65* |  | - | *0.73* |  | - | *0.49* |  | - | *0.25* |
| **Outlier-corrected Effect** | N/A | *N/A* |  | 0.87  (0.54 - 1.42) | *0.61* |  | N/A | *N/A* |  | N/A | *N/A* |  | N/A | *N/A* |  | N/A | *N/A* |

Abbreviation: IVW, Inverse Variance Weighted regression analysis; RE, Random effect; FE, Fixed effect; OR, Odds ratios; CI, Confidence interval; BCa, bladder cancer; RCC, renal cell carcinoma; PCa, prostate cancer; BCa, bladder cancer; RCC, renal cell carcinoma; PCa, prostate cancer.

Significant results (p<0.05; tested two-sided) are shown in bold. Odds ratios represent the odds of urological cancers (BCa, RCC and PCa) for lifetime cannabis users versus non-users (when cannabis is the exposure) or the odds of lifetime cannabis use for those with a urological cancer diagnosis versus those without (when cancer is the exposure) (OR).

**Figure S1**


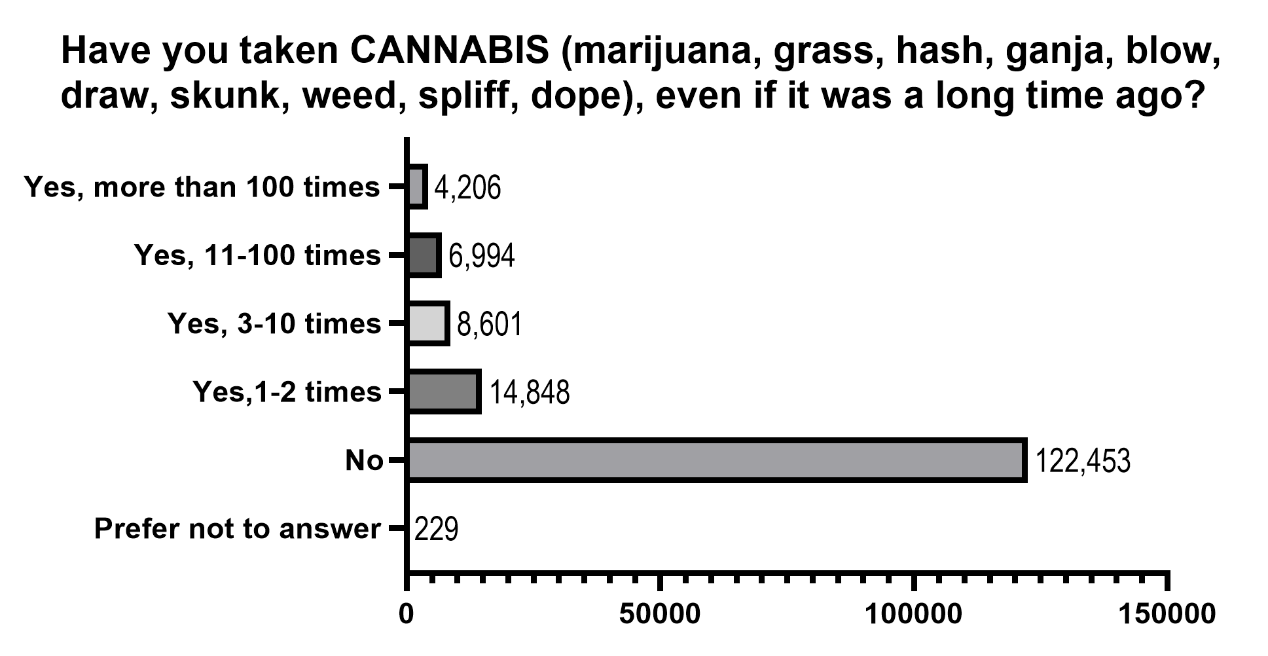


The standard questionnaire of UKB about cannabis use.

**Figure S2**


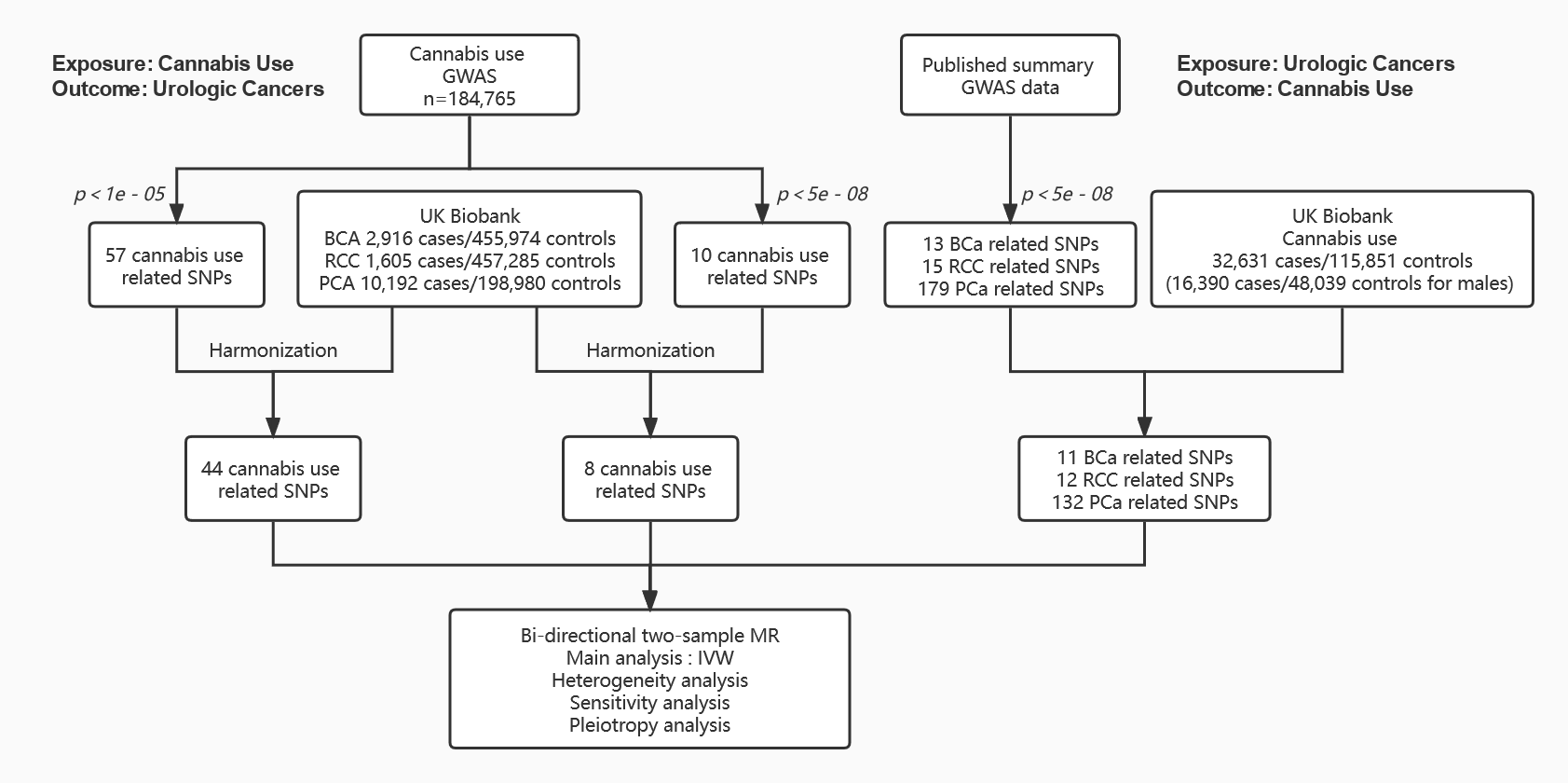


Flowchart showing the process of bi-directional two-sample MR.

Abbreviation: BCa, bladder cancer; RCC, renal cell carcinoma; PCa, prostate cancer; BMI, body mass index; SNP, single nucleotide polymorphism.

**Figure S3**


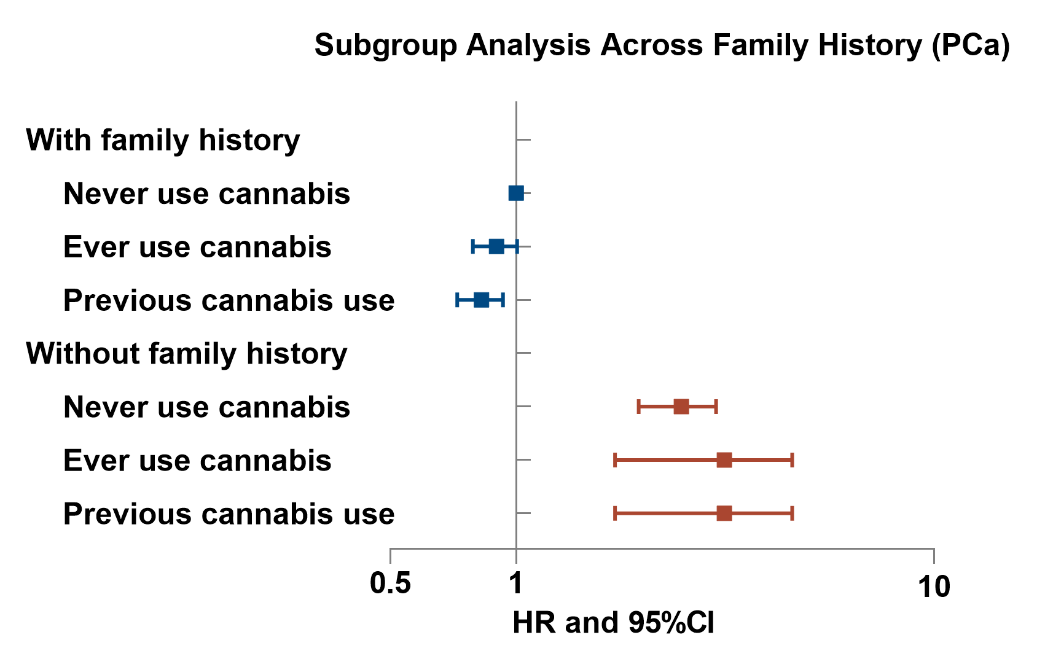


Forest plot: HRs of cannabis use in subgroups of different family history for PCA.

Abbreviation: PCa, prostate cancer; HR, hazard ratio; CI, confidence interval.

**Figure S4**


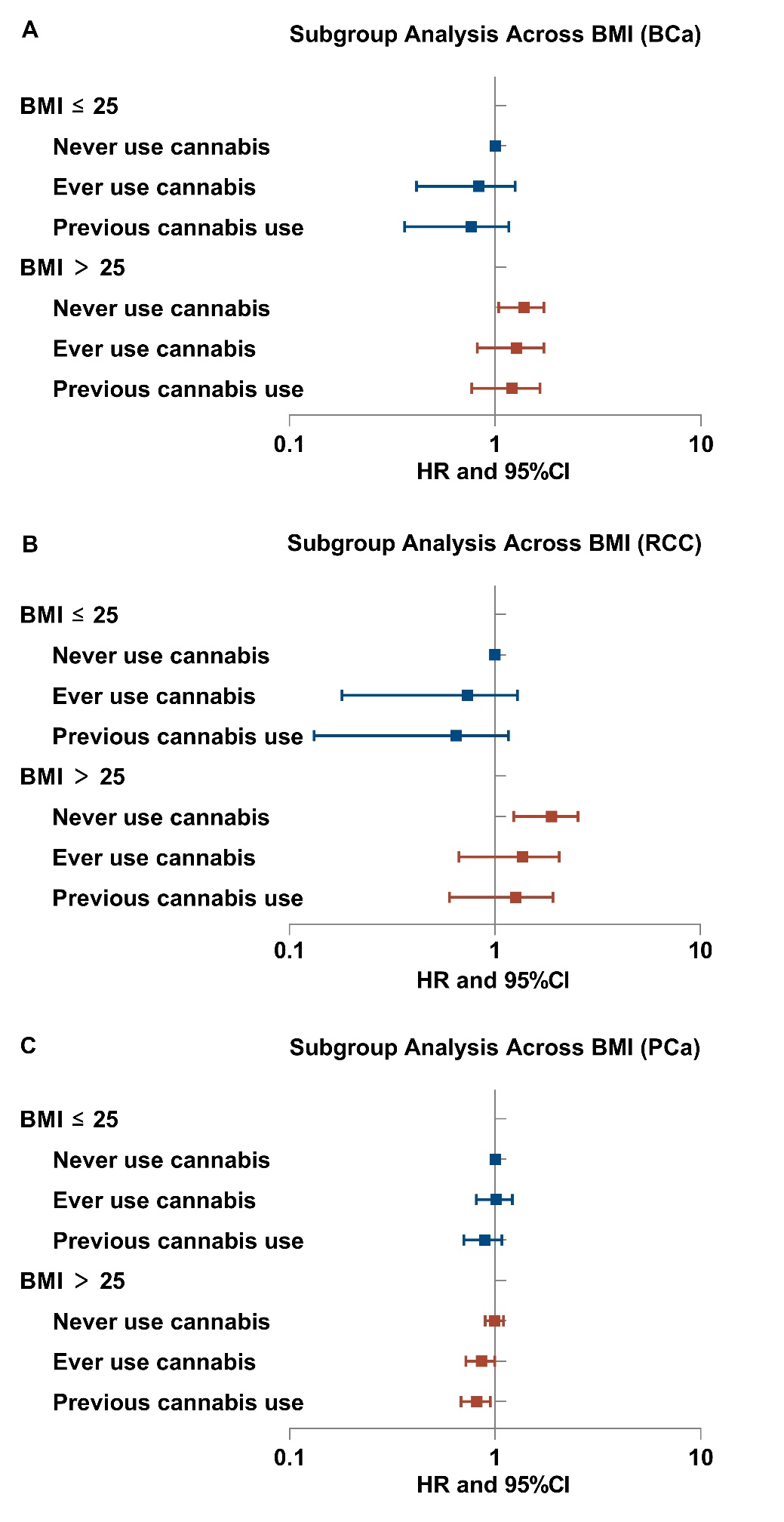


Forest plot: HRs of cannabis use in subgroups of different BMI (A) for BCa; (B) for RCC; (C) for PCa.

Abbreviation: BCa, bladder cancer; RCC, renal cell carcinoma; PCa, prostate cancer; BMI, body mass index; HR, hazard ratio; CI, confidence interval.

**Figure S5**


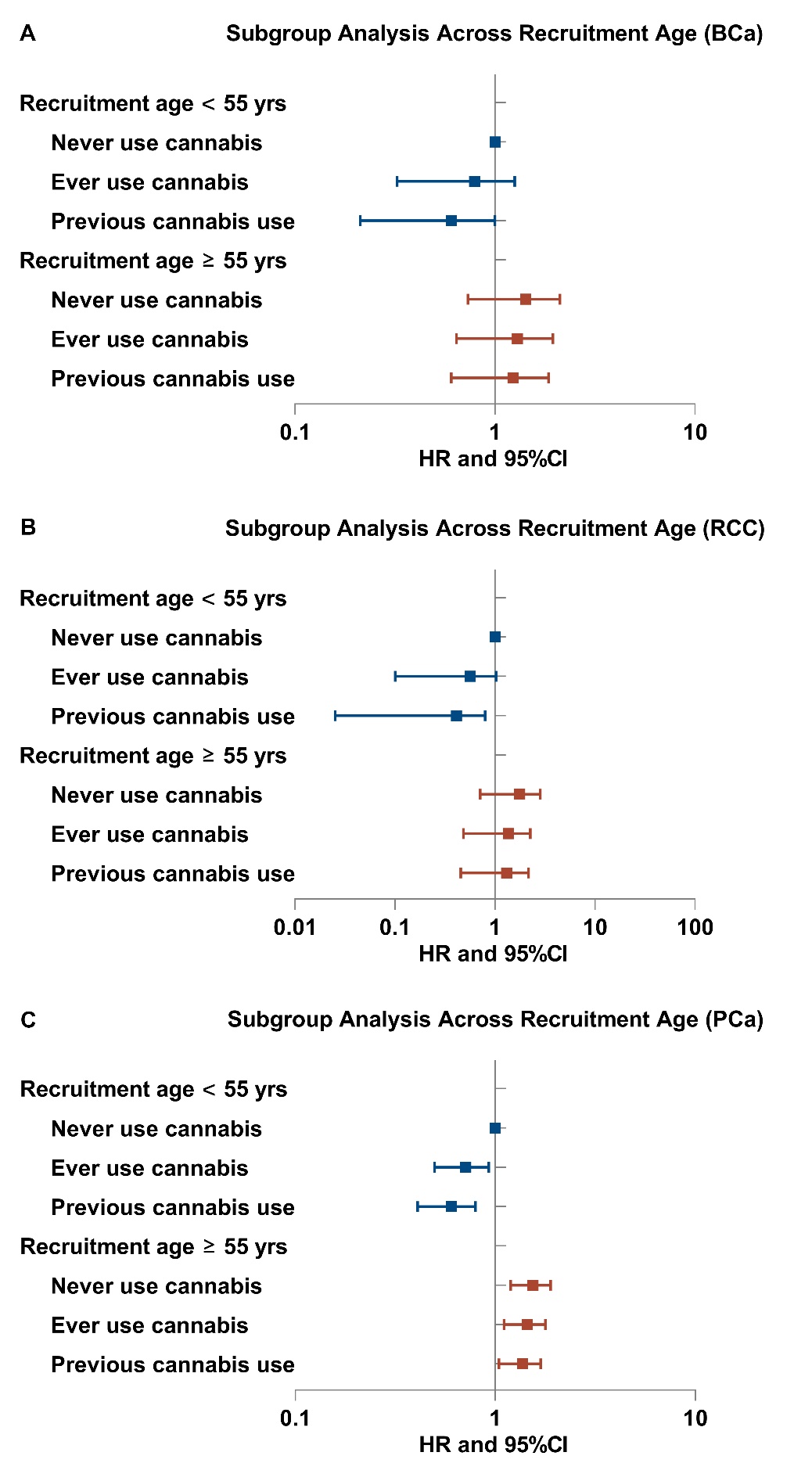


Forest plot: HRs of cannabis use in subgroups of different recruitment age (A) for BCa; (B) for RCC; (C) for PCa.

Abbreviation: BCa, bladder cancer; RCC, renal cell carcinoma; PCa, prostate cancer; HR, hazard ratio; CI, confidence interval.

**Figure S6**


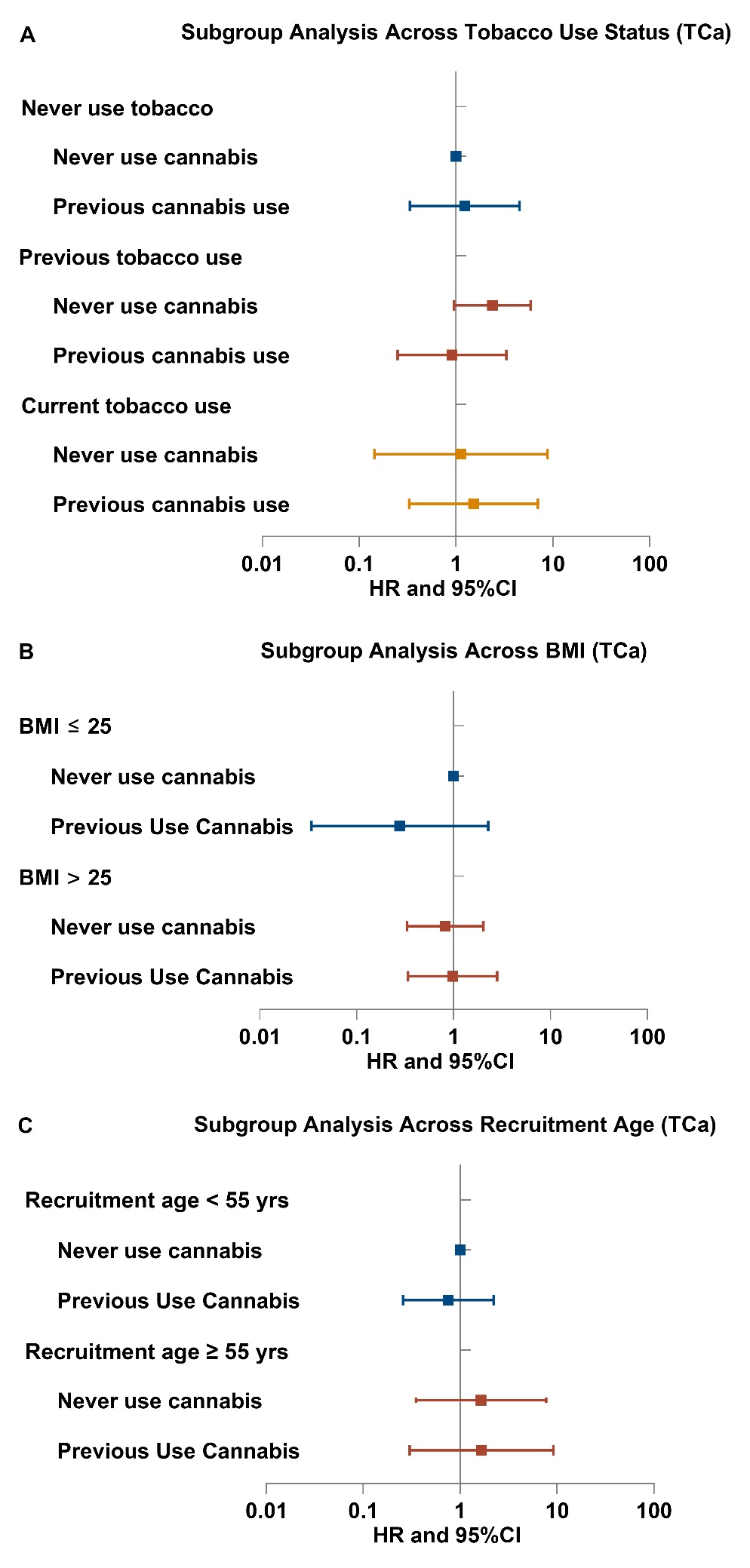


Forest plot showing HR of cannabis use for TCa in subgroups (A) of recruitment age; (B) of tobacco use status; (C) of BMI.

Abbreviation: TCa, testicular cancer; BMI, body mass index; HR, hazard ratio; CI, confidence interval.

**Figure S7**


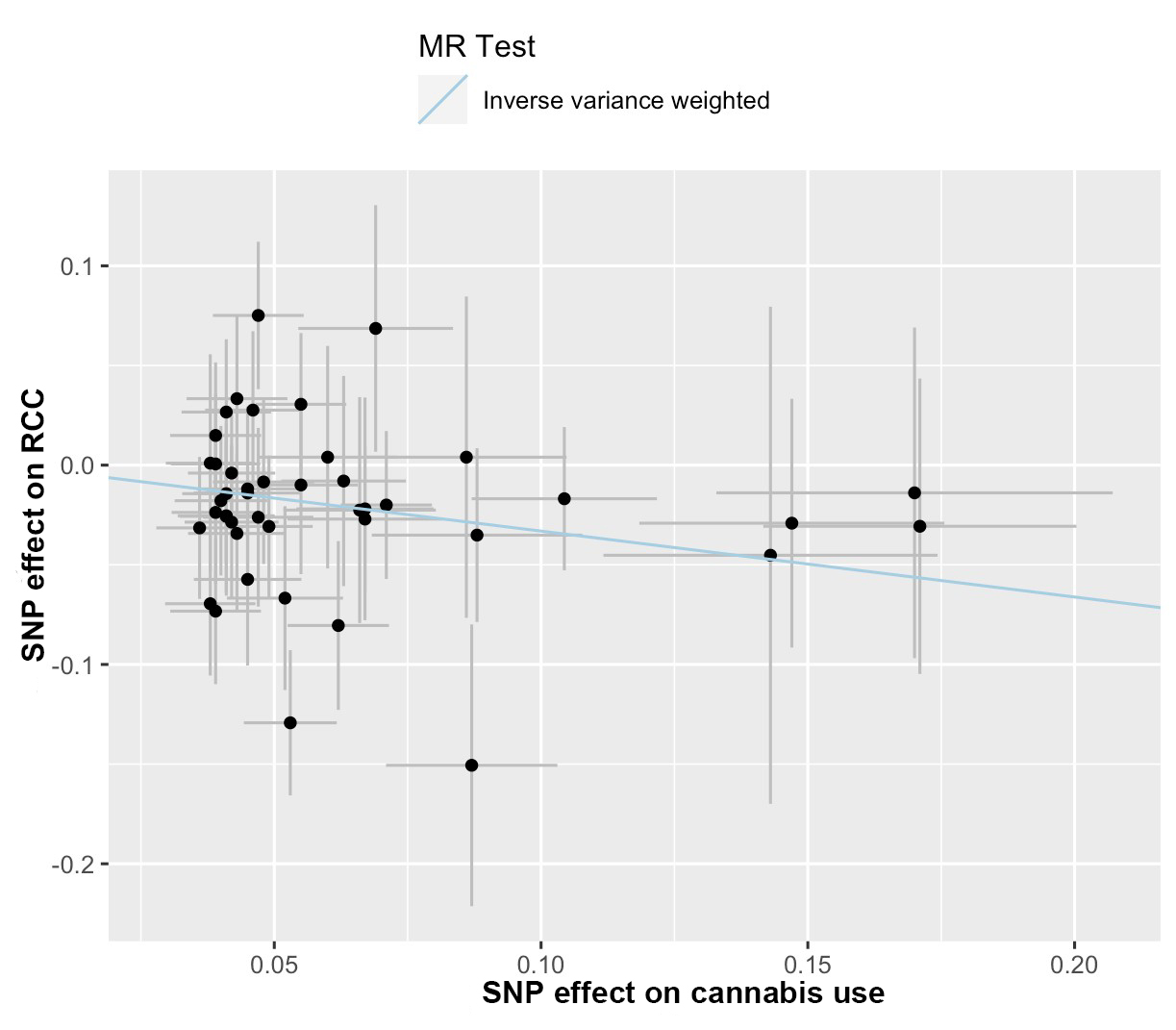


Scatter plot to visualize causal effect of cannabis use on RCC. The slope of the straight line indicates the magnitude of the causal association.

Abbreviation: IVW indicates inverse-variance weighted; MR, Mendelian randomization; RCC, renal cell carcinoma.

**Figure S8**


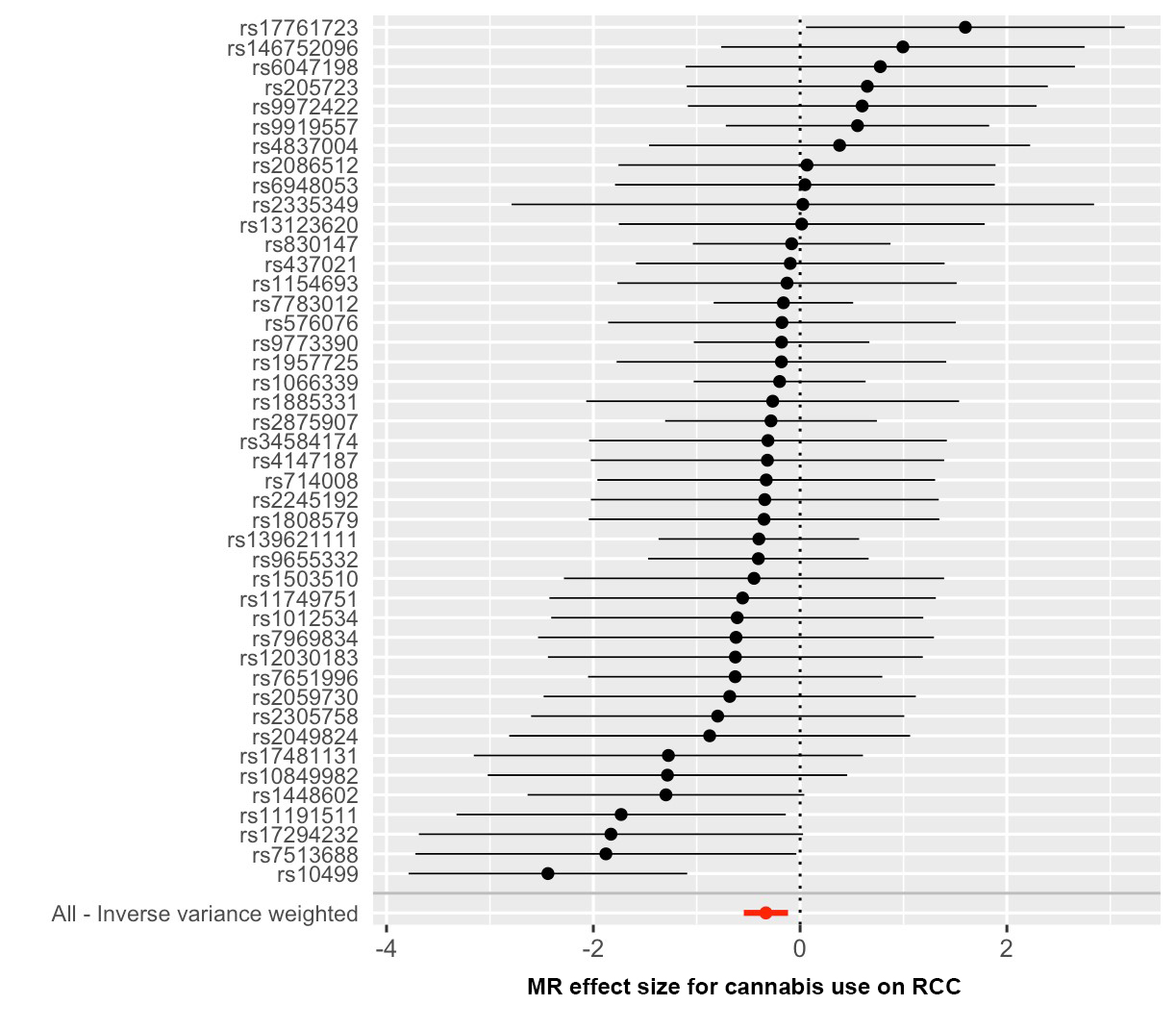


Forest plots depicting results of two-sample MR for the effect cannabis use on RCC.

Abbreviation: MR, Mendelian randomization; RCC, renal cell carcinoma.

**Figure S9**


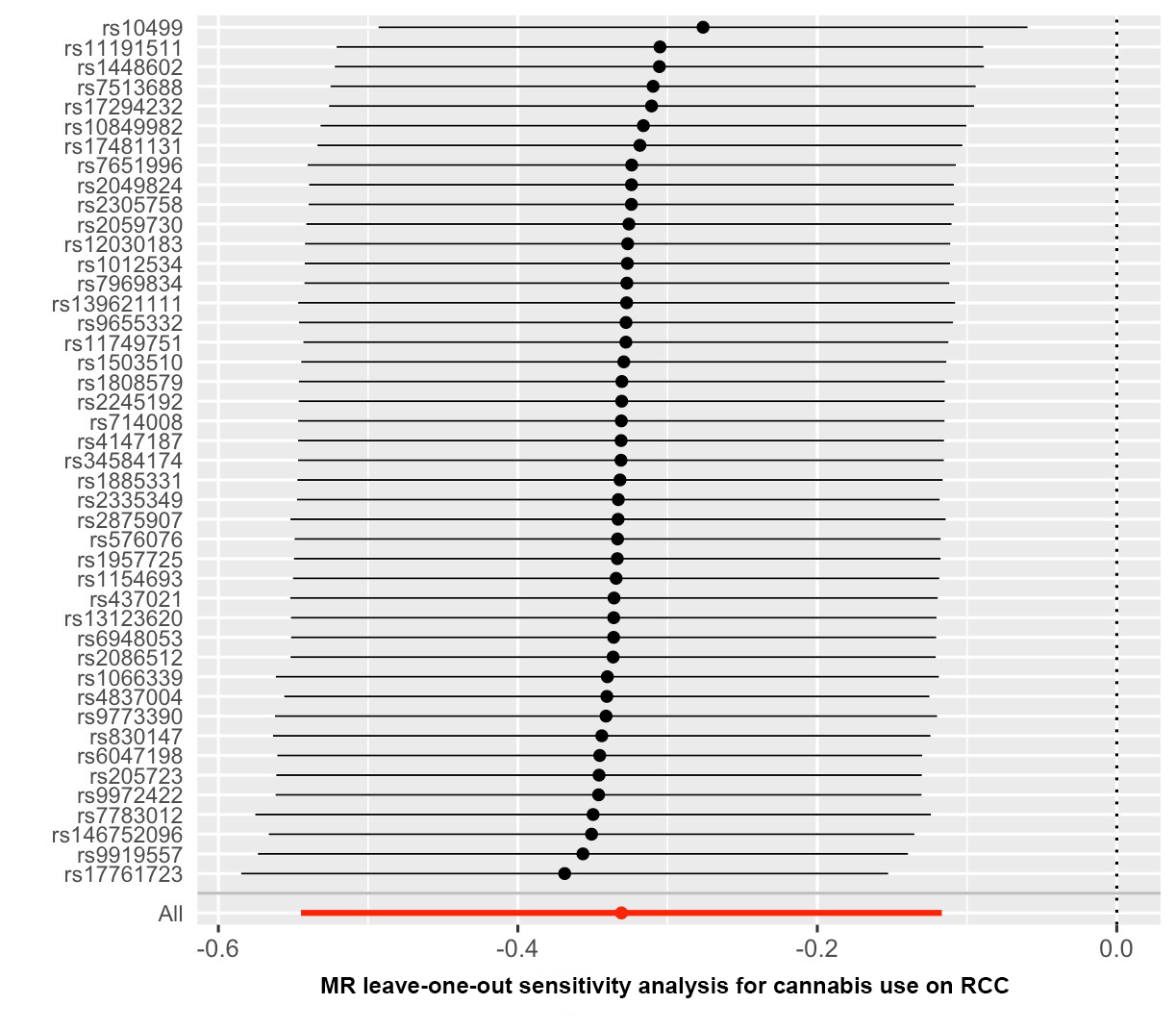


Forest plots depicting results of leave-one-out sensitivity analysis of MR for the effect cannabis use on RCC.

Abbreviation: MR, Mendelian randomization; RCC, renal cell carcinoma.

**Figure S10**


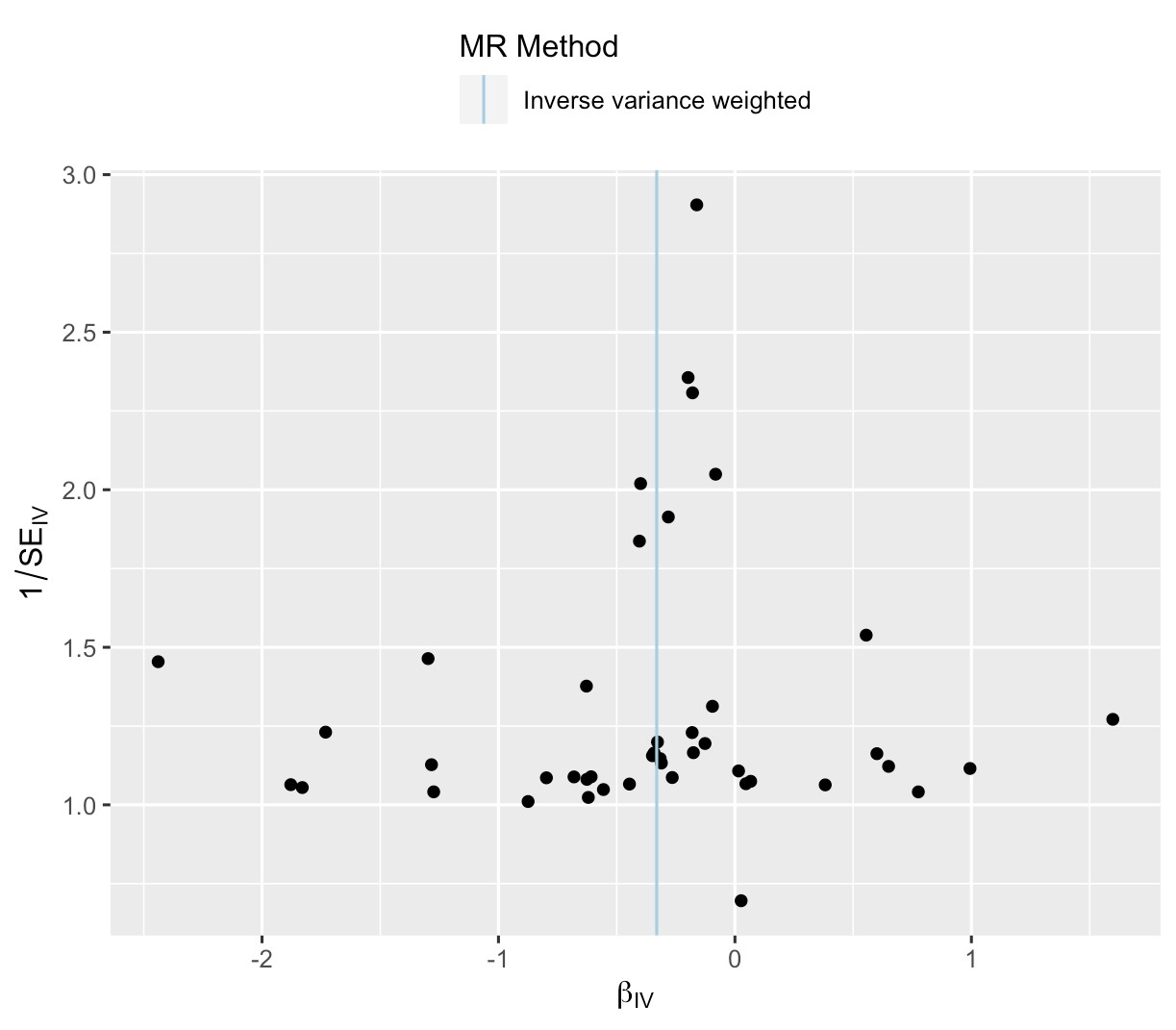


Funnel plots to visualize heterogeneity of MR estimates for the effect cannabis use on RCC.
